# Evidence for publicly reported quality indicators in residential long-term care: a systematic review

**DOI:** 10.1186/s12913-022-08804-7

**Published:** 2022-11-24

**Authors:** Magdalena Osińska, Lauriane Favez, Franziska Zúñiga

**Affiliations:** Institute of Nursing Science, University of Basel, Bernoullistrasse 28, 4056 Basel, Switzerland

**Keywords:** Nursing homes, Long-term care, Quality indicators, Public reporting of healthcare data, Review

## Abstract

**Background:**

An increasing number of countries are using or planning to use quality indicators (QIs) in residential long-term care. Knowledge regarding the current state of evidence on usage and methodological soundness of publicly reported clinical indicators of quality in nursing homes is needed. The study aimed to answer the questions: 1) Which health-related QIs for residents in long-term care are currently publicly reported internationally? and 2) What is the methodological quality of these indicators?

**Methods:**

A systematic search was conducted in the electronic databases PubMed, CINAHL and Embase in October 2019 and last updated on August 31st, 2022. Grey literature was also searched. We used the Appraisal of Indicators through Research and Evaluation (AIRE) instrument for the methodological quality assessment of the identified QIs.

**Results:**

Of 23′344 identified records, 22 articles and one report describing 21 studies met the inclusion criteria. Additionally, we found 17 websites publishing information on QIs. We identified eight countries publicly reporting a total of 99 health-related QIs covering 31 themes. Each country used between six and 31 QIs. The most frequently reported indicators were pressure ulcers, falls, physical restraints, and weight loss. For most QI sets, we found basic information regarding e.g., purpose, definition of the indicators, risk-adjustment, and stakeholders’ involvement in QIs’ selection. Little up to date information was found regarding validity, reliability and discriminative power of the QIs. Only the Australian indicator set reached high methodological quality, defined as scores of 50% or higher in all four AIRE instrument domains.

**Conclusions:**

Little information is available to the public and researchers for the evaluation of a large number of publicly reported QIs in the residential long-term care sector. Better reporting is needed on the methodological quality of QIs in this setting, whether they are meant for internal quality improvement or provider comparison.

**Supplementary Information:**

The online version contains supplementary material available at 10.1186/s12913-022-08804-7.

## Background

Due to demographic changes, residential long-term care (RLTC) institutions are increasingly challenged by growing numbers of older residents with complex care needs and dwindling supply of trained workforce [1]. The share of people aged over 80 years is expected to double by 2050 [2]. Simultaneously the number of RLTC workers per 100 people aged 65 and over has stagnated or decreased in many countries, raising concerns about capacity to meet the care needs in the coming years [3]. These challenges make monitoring and assessing of quality in RLTC crucial, where we understand RLTC as any type of setting where older adults reside and receive 24 h formal long-term care services, i.e., by paid care staff, and there is an expectation of a long stay [4].

Considerable public and private spending on health care results in an increased demand for transparency and accountability for the quality of care provided [2, 5]. Accordingly, quality indicators (QIs) are increasingly used internationally to measure, report and track quality of care over time. In the US, for example, the basis for the nation-wide measurement was laid with the 1987 Omnibus Budget Reconciliation Act (OBRA) – reacting to reports of quality problems in RLTC –, which mandated a comprehensive assessment with a Minimum Data Set (MDS). Based on the MDS, an array of QIs were developed, tested and implemented to initiate quality improvement in the context of the Nursing Home Case Mix and Quality Demonstration Project of the Centers of Medicare & Medicaid Services (CMS) starting in 1996 and linking funding schemes to QI measurement [6, 7]. QIs can be used to identify potential quality problems or to guide quality improvement initiatives [8, 9]. Their public reporting allows benchmarking between health care institutions or against established thresholds. Therefore, QIs can be a valuable source of information for health care providers, residents, insurers, governments and researchers. Although QIs are not absolute measures of quality, they can reflect aspects of it by describing desired or undesired structures, processes and outcomes [9]. QIs need to meet minimum methodological standards to be usable and useful. They need to be carefully developed to ensure that they accurately and consistently measure what they are supposed to measure. In order to be accepted as valid measures [10], QIs should cover relevant subjects and be feasible for use in practice. If intended for provider comparison, it is important that they show sufficient discriminative power and that risk-adjustment is applied to account for between-provider differences, e.g., in residents’ case-mix [11]. Finally, QIs need to be comprehensible to the public, so that the information can be correctly interpreted. Considering its importance, the information regarding the methodological quality of QIs should be publicly available to allow for correct interpretation of the results and international comparisons. This includes information about their development, definitions, measurement, risk-adjustment and measurement quality (e.g., its validity, reliability, and discriminative power).

Two published reviews considered methodological quality of QIs used in RLTC. In 2009, Nakrem at al [12]. investigated the development descriptions and the validity testing of national QIs obtained from peer-reviewed and grey literature in a convenience sample of seven countries. This review included indicators considered nursing sensitive, but not necessarily publicly reported. Hutchinson at al. (2010) [13] systematically examined the evidence for the validity and reliability of QIs based on Resident Assessment Instrument - Minimum Data Set (RAI-MDS) 2.0. Although several countries derive their QIs from RAI-MDS data, both the publicly reported indicators from RAI-MDS and the instrument itself have evolved over the last 10 years.

With the launching of national QIs in 2019, Switzerland is one of the countries that has recently started to measure quality in RLTC and the results have recently been published for the first time. A total of 6 QIs were included in the initial QI set, which is expected to be expanded [11]. Since an increasing number of countries are using or planning to measure quality in RLTC, knowledge regarding the current state of evidence on the usage and quality of publicly reported health-related QIs is needed. By health-related QIs we understand indicators concerning the process of health-care (e.g., medication review) or clinical outcomes of the residents, such as pain or falls. In order to inform choices regarding possible themes of QI measurements and to provide an update on the current state of evidence, this systematic literature review aimed to answer the following questions: 1) Which health-related QIs for RLTC are currently publicly reported internationally? and 2) What is the methodological quality of these indicators?

## Methods

### Search strategy

A systematic search was conducted in the electronic databases PubMed, CINAHL and Embase in October 2019 and updated twice, last on August 31st 2022. We constructed a search string including Medical Subject Headings (MeSH) and key words related to the concepts of RLTC and QIs or public reporting, connected with appropriate Boolean operators (see Additional file 1). We also tracked references of included articles for unidentified relevant studies. Additionally, we searched for grey literature on organizational websites mentioned in identified studies, as well as on websites recommended by experts or identified when screening published reports. Grey literature was searched in English, German, French, and Spanish and an electronic translation to screen websites in other languages was used. If the published literature on publicly reported QIs was scarce or unclear, we contacted the reports’ authors or website owners via e-mail for further information. Additional file 2 provides a list of consulted websites and contacted institutions.

### Inclusion and exclusion criteria

Based on preliminary inclusion and exclusion criteria, the first author screened around 40 papers for title and abstract to refine the criteria. In the discussion with the last author, the final set of criteria was developed, with refinements in the definition of RLTC (e.g., no short-stay residents) and target population (e.g., only QIs including all residents and not subgroups like palliative care or rehabilitative care). In the end, we included published primary studies and grey literature (e.g., government reports) which described the development, testing or measurement of publicly reported health-related QIs in RLTC. The QIs were included, if we found information that they were publicly reported at the time of the review at any level (e.g., facility, regional, national) on mandatory or voluntary basis. The QIs had to be related to the process of health-care or to health-related resident outcomes. We excluded QIs related to quality of life, such as satisfaction with care services, structural QIs, such as staffing levels or financing, and QIs designed specifically for short-term or specialized care, such as rehabilitative or palliative care, since the latter cannot be applied to the general population of residents. We also excluded editorials, comments and reader’s letters.

### Screening and data extraction

Identified studies were entered and managed in EndNote and duplicates were removed. One author (MO) screened by title and abstract for relevance according to the inclusion criteria and non-eligible studies were removed. Potentially eligible articles underwent full-text screening (MO) for inclusion. Doubts about inclusion were clarified with the third author (FZ). Reference lists of included studies were also screened (MO) for further relevant literature. Eligible grey literature (e.g., reports and manuals) found on websites in other languages than English, German, French, or Spanish were translated into English with DeepL Pro or Google Translate translation service before being screened (MO, FZ). Definitive inclusion was agreed on by two authors (MO and FZ).

### Methodological assessment

We used the Appraisal of Indicators through Research and Evaluation (AIRE) instrument for the methodological assessment of the identified QIs [14]. AIRE is a validated instrument for the critical appraisal of QIs and has been previously used in various settings [15–18]. AIRE includes 20 items in four domains: 1) purpose, relevance and organizational context, 2) stakeholder involvement, 3) scientific evidence, and 4) additional evidence, formulation and usage. A detailed description of the items can be found in Additional file 3. All three authors appraised the QIs independently using information from included studies, websites and emails from consulted organisations. The assessment of each AIRE item is based on a 4-point Likert scale, ranging from 1 ‘strongly disagree or no information available’ to 4 ‘strongly agree’. Differences of more than 1 point in assessment were resolved in discussion between the authors. We calculated standardized scores per domain, which can range from 0 to 100%, with a higher score indicating a higher methodological quality.

## Results

### Search results

**Fig. 1:**
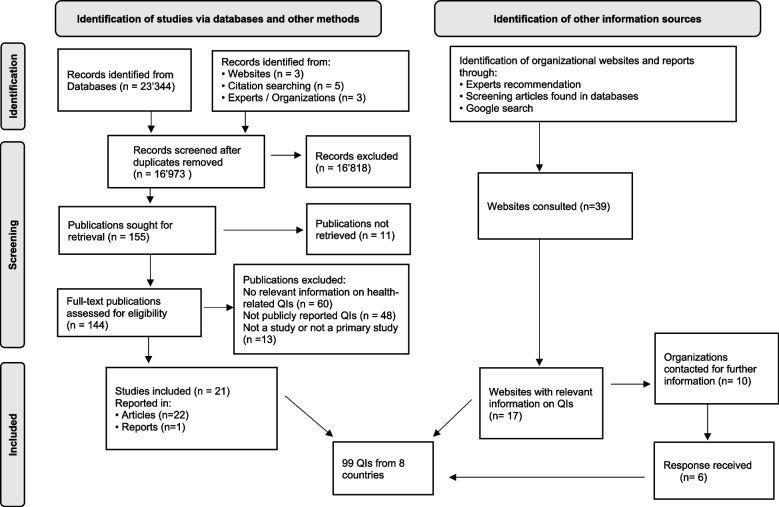
Flow diagram of QI selection

The systematic review – including records found in other sources – identified 23′344 potential records. After removing duplicates, 16′973 were screened by title and abstract, 144 publications underwent full-text screening and 23 met inclusion criteria. Of 39 consulted websites 17 contained information on QIs relevant for the review. We contacted 10 organizations for further information, of which 6 replied. Figure 1 presents the flow diagram for the QI selection process and Additional file 4 gives an overview of publications excluded based on full-text screening.

### Description of included studies and grey literature

Twenty two articles [6, 8, 19–38] and one study report [39] were included. The articles were published between 1995 and 2021. Nineteen studies were conducted in the U.S., one in Canada [23] and one in the Netherlands [21]. Eleven studies described the development or evaluation of QI sets and the other evaluated single QIs or QIs pertaining to one theme. Apart from the study from the Netherlands [21], all studies regarded QIs derived from the MDS. The included studies are described in Table 1.

**Table 1:** Description of included studies

Author (year)	Country	Aim	Study population (sample size)	Evaluated QI(s)	Methods and measurements
Bates-Jensen et al. (2003) [19]	U.S.	To determine whether nursing homes that score better on pressure ulcer prevalence MDS QI provide different pressure ulcer care.	329 residents at risk for pressure ulcer development in 16 nursing homes	Pressure ulcers	Sixteen QIs regarding pressure ulcer care, nutrition and incontinence management were scored using medical record data, direct human observation, interviews, and data from wireless thigh movement monitors.
Berg et al., (2002) [20]	U.S.	To summarize work done to identify and evaluate QIs for LTC settings.	MDS assessments from 1995 to 1997 from all nursing facilities in 5 states	143 QIs	Review of existing QIs, preliminary analyses that examined the incidence and prevalence of selected QIs in nursing facilities and the stability of the QI rates over time.
Bours et al. (1999) [21]	Netherlands	To describe the development of a national registration form to measure the prevalence of pressure ulcers.	34 experts in Delphi panel; 1 hospital, 1 nursing home and 1 home health care agency	Pressure ulcers	Literature review and Delphi panel, a pilot study with interrater reliability and feasibility assessment
Cadogan et al. (2004) [22]	U.S.	To compare pain-related care processes between nursing homes that scored high and low on the pain prevalence MDS QI	255 residents in 16 nursing homes (8 with low and 8 with high pain prevalence)	Pain	Care processes related to pain assessment, documentation, and treatment were independently evaluated by trained research staff using standardized resident interview and medical record review protocols.
Estabrooks et al. 2013 [23]	Canada	To identify RAI-MDS 2.0 QIs believed to be the most sensitive to clinical practice.	2 experts on QI and 16 informants (physicians, nurses and decision/ policy-makers)	35 MDS 2.0. QIs	Thirteen QIs chosen by the experts were rated individually by the informants for overall “practice sensitivity”. The informants were also asked to identify the domain to which the QI was most sensitive (nursing care, physician care, or policy maker).
Hill-Westmoreland & Gruber-Baldini, 2005 [24]	U.S.	To assess the agreement between falls as recorded in the MDS and fall events abstracted from chart documentation.	462 residents in 56 nursing homes	Falls	Falls were abstracted from resident charts and compared with MDS falls variables: fell in the past 30 days and fell in the past 180 days.
Jones et al., 2010 [25]	U.S.	To describe a method for adjustment of nursing home QIs defined using the MDS.	5738 residents in 209 nursing homes; MDS data from 3294 U.S. facilities and 92 Canadian facilities.	79 MDS QIs	Development of new risk adjustment, assessment of validity and stability of QIs over time.
Karon et al., 1999 [26]	U.S.	To examine the stability of MDS QIs over each of two 3-month periods and one 6-month periods.	512 nursing facilities	30 MDS QIs	QI stability was assessed using corelations or Cohen’s Kappa. The variables included: proportion of residents in the facility with the QI condition; the facility’s percentile rank in its state; and a variable indicating whether the facility’s rank exceeded the 90th percentile in its state.
Mintz et al., 2021 [35]	U.S.	To validate the MDS v3.0 items on falls and injuries with chart review.	251 residents in 2 nursing homes	Falls with major injury	Fall and injury agreement between the MDS and chart review was assessed with Cohen’s Kappa test. Sensitivity, specificity and positive predictive value were calculated.
Mor et al., 2003 [27] Morris et al., 2003 [39]	U.S.	To assess inter-rater reliability of MDS assessments which generate the data used for publicly reported QIs. To report on validation of long-term and post-acute care QIs.	5758 residents in 209 nursing facilities in 6 states	22 MDS QIs 45 MDS QIs	Resident assessments by facility nurses and research nurses were compared using Kappa statistics.
Phillips et al., 2007 [36]	U.S.	To assess the impact of facility and resident characteristics on ADL change.	36′584 residents in 1334 nursing homes	Change in ADL function	Multivariate models estimated at the individual level.
Rantz et al., 1997 [29]	U.S.	To verify the accuracy of QIs derived from MDS data	10 nursing homes	14 QIs	Comparison of facilities performing well with those performing poor on QIs.
Rantz et al., 2004 [28]	U.S.	To examine cost, staffing, and quality of care information from the MDS and Medicaid cost report.	92 nursing homes	23 MDS QIs	Facilities were grouped based on how well they performed on the MDS QIs into good, average and poor. Stability of facility performance and sensitivity of QIs to discriminate between groups was analysed.
Sanghavi et al., 2020 [37]	U.S.	To assess the accuracy of nursing home self-report of major injury falls on the MDS.	MDS assessments and Medicare claims data 2011–2015	Falls with major injury	The proportion of claims-identified falls reported for each fall-related MDS item and the correlation between fall rates based on claims vs the MDS was calculated.
Schnelle et al., 2004 [30]	U.S.	To investigate whether the use of restraint differs in nursing homes that score in the upper and lower quartiles on the MDS prevalence of restraint QI.	413 residents in 14 nursing homes	Restraint	Eight care processes related to the management of restraints, gait and balance problems were defined and operationalized into clinical indicators. Research staff conducted direct observations to determine the prevalence of restraining devices and identify resident and staff behaviours that may be affected by restraint use.
Schnelle et al., 2003 [31]	U.S.	To determine if nursing homes that score in the lower 25th percentile versus the upper 75th percentile on MDS incontinence QIs provide different incontinence care processes.	347 long-term residents in 14 skilled nursing facilities for the MDS “prevalence of incontinence” indicator and 432 residents in 16 skilled nursing facilities for the MDS “prevalence of incontinence without a toileting plan” indicator.	Incontinence	Nine care processes related to incontinence were defined and operationalized into clinical indicators. Research staff assessed implementation of each care process on 3 consecutive days. The assessment included resident interviews, physical performance evaluations, and chart abstraction using standardized protocols
Simmons et al., 2003 [34]	U.S.	To determine whether nursing homes that score differently on prevalence of weight loss, according to MDS QI, also provide different processes of care related to weight loss.	400 long-term residents in 16 nursing homes	Weight loss	Sixteen care processes related to weight loss were defined and operationalized into clinical indicators. Research staff conducted measurement of nursing home staff implementation of each care process during assessments on three consecutive days, which included direct observations during meals, resident interviews, and medical record abstraction using standardized protocols.
Stevenson et al., 2004 [32]	U.S.	To determine the validity of the MDS to detect cases of urinary tract infection (UTI) that meet specific evidence-based criteria.	16 nursing homes	Urinary tract infection	Data from prospective surveillance of all types of infection, including UTI, and data on clinical manifestation, microbiology, and treatment were compared with MDS data on identification of UTI.
Wu et al., 2005 [33]	U.S.	To examine facility variation in data quality of the level of pain documented in the MDS as a function of level of hospice enrolment in nursing homes.	3469 nonhospice residents from 178 nursing homes	Pain	Study nurses’ and nursing home staff’s pain rating was compared across nursing homes with high, medium, or low hospice use. Multilevel models were built to assess the effect of nursing home hospice use levels on the occurrence of false positive and false negative errors in nursing home-rated “severe pain”.
Wu et al., 2009 [38]	U.S.	To examine the association between measurement errors in 8 MDS-derived measurement scales and 1) the characteristics of residents and nursing homes, 2) the MDS-derived QIs.	5174 pairs of MDS assessments from 206 nursing homes	Pressure ulcers Cognitive function Incontinence Restraints Pain ADL	Multivariate multilevel models were used to identify nursing home and resident characteristics associated with the data quality of MDS QIs. Coding differences between nursing home staff and study nurses served as outcomes. The pattern between the state averages of QIs and predicted averages of measurement bias was investigated.
Zimmerman et al., 1995 [6] Zimmerman, 2003 [8]	U.S.	To report on the development and testing of QIs from RAI-MDS data.		31 of initial 175 QIs were pilot-tested, and 24 were included in a final set.	Clinical review, pilot testing of accuracy, feasibility and predictive power, description of risk adjustment.

*Abbreviations*: *ADL* Activities of Daily Living, *MDS* Minimum Data Set, *QI* Quality indicator, *RAI* Resident Assessment Instrument, *U.S*. United States, *UTI* Urinary tract infection

All countries published information on their QIs on websites [40–56]. There we found manuals or guides on QI definitions, measurements, collection, reporting and interpretation in the U.S. [57–59], Canada [60–75], Australia [76, 77], New Zealand [78], the Netherlands [79], Belgium [80], Norway [81–87] and Sweden [88–91]. Development and validation reports were available for the QI sets from the U.S. [39], Canada [92] and Australia [93]. Some information on QI selection and development was also included in documents from the Netherlands [79], Belgium [80] and Sweden [94]. QI results from all countries were published on websites accessible to the public [42, 45, 47–50, 52, 53, 55, 56, 95].

### Purpose, data collection and reporting characteristics of the QI sets

We identified eight countries publicly reporting health-related QIs. They are selected, collected and reported in different ways based on feasibility, practicality and stated purposes. In the U.S., Canada, New Zealand, Norway and Sweden all or most QIs are built from already available data (i.e., routine assessments or national registries), whereas Australia, Belgium and the Netherlands use separate collection methods (e.g., surveys). Most countries provide online manuals with guidance on how data should be collected; e.g., in Australia, QI data is to be collected via full body assessment (pressure ulcers) or auditing charts (e.g., falls) quarterly by filling out a sheet per resident. Data is then entered in an online platform aggregated at facility level [77]. However, in the manuals we consulted we did not find information about, e.g., minimal standards for training on data collection. Most countries state several purposes for their QI sets, but some differences in their focus can be observed. The U.S. and Canada focus the most on comparing individual service providers. Australia, Belgium and Sweden concentrate rather on supporting RLTC institutions in monitoring and improving care [45, 52, 76, 80]. The main purpose of the QIs in the Netherlands is stimulating learning and improvement in care teams [79]. QIs in Norway are aimed for meeting the information needs of the healthcare sector and the government (e-mail communication).

The purpose of the QIs influences the level (i.e., national, regional, facilities) and the manner of reporting. For instance, the U.S. and Canada developed rating systems and specifically dedicated websites intended to make comparison easier for consumers and almost only use resident outcome indicators (e.g., prevalence of falls) [50, 95]. Countries focusing rather on supporting RLTC institutions in improving care than on provider comparison use more process indicators (e.g., medication review) and do not risk-adjust QIs. Table 2 presents an overview of data collection methods, measurement frequency and reporting characteristics. Types (i.e., prevalence or incidence) and measurement level (i.e., process or outcome) of included QIs are described in Additional file 3.

**Table 2:** Data collection, measurement and reporting of QIs from each country

Country	Type of data collection	Data source	Measurement frequency	Reporting frequency	Website	Lowest level of publicly reported data	Risk-adjustment
US	routine	MDS 3.0, CMS claims	quarterly	yearly	Medicare.gov	facility	Partly
CA	routine	RAI-MDS 2.0	quarterly	yearly	Your Health System	facility	Yes
NZ	routine	interRAI LTCF	quarterly	quarterly	interRAI New Zealand	District Health Board	No
AU	separate	survey	quarterly	quarterly	GEN aged care data	region	No
BE	separate	survey	yearly	yearly	Vlaams Instituut voor Kwaliteit van Zorg	region	No
NL	separate	survey	yearly	yearly	Zorginstituut Nederland	facility	No
NO	routine, separate	Registers / survey	yearly/ half-yearly	yearly/ half-yearly	Helsedirektoratet	municipality	No
SE	routine	Registers/survey	yearly	yearly	Kolada Senior Alert Socialstyrelsen Folkhälsomyndigheten	municipality	No

*Abbreviations*: *AU* Australia, *BE* Belgium, *CA* Canada, *CMS* Centers for Medicare & Medicaid Services, *LTCF* Long-Term Care Facilities, *MDS* Minimum Data Set, *NL* Netherlands, *NO* Norway, *NZ* New Zealand, *QI* Quality Indicator, *RAI* Resident Assessment Instrument, *SE* Sweden, *US* United States

### Publicly reported health-related QIs

In the identified eight countries, a total of 99 QIs were publicly reported, covering 31 themes. Each country reported between six and 31 health-related QIs. The most frequently reported indicators were related to pressure ulcers, falls, physical restraints and weight loss. Table 3 presents the identified QIs themes. In some cases, more than one indicator is used for the same theme. A full list of all included indicators per country can be found in Additional file 3.

**Table 3:** Themes covered by the publicly reported QIs

Indicator theme	US	Canada	New Zealand	Australia	Belgium	Nether-lands	Norway	Sweden	No. of reporting countries
**Functional ability**									
Communication			x						1
Cognitive ability			x						1
Mobility	x	x^a^	x						3
Self-care	x		x						2
**Clinical situations**									
Continence		x^a^	x			x		x^e^	4
Indwelling catheter	x		x						2
Urinary tract infection	x		x						2
Nosocomial infections							x	x^f^	2
Weight loss	x		x	x^c^	x			x^e^	5
Feeding tube			x						1
Food preferences						x			1
Nutrition assessment							x		1
Oral health								x^e^	1
Physical restraints	x	x^a^	x	x^c^	x	x			6
Fall	x	x^a^	x	x^d^	x			x^e^	6
Pain		x^a^	x						2
Pressure ulcers	x	x^a^	x	x^c^	x	x		x^e^	7
**Psychosocial aspects**									
Signs of depression		x^a^	x						2
Behaviour change		x^a^	x						2
**Pharmacotherapy**									
Antianxiety or hypnotic medication	x								1
Antipsychotics	x	x^a^						x^g^	3
Antibiotics							x	x^f^	2
Polypharmacy				x^d^				x^g^	2
Medication review						x	x		2
Inappropriate medication								x^f^	1
Medication errors					x				1
**Health services**									
Hospitalisation/emergency ward visit	x	x^b^							2
Medical/dental examination, medical treatment hours							x		1
Influenza/ pneumococcal vaccination	x								1
Advance care plan					x	x			2
Death in nursing home					x				1

QI published by

^a^CIHI (Canadian Institute for Health Information)

^b^Health Quality Ontario

^c^Australian Government Department of Health

^d^Victoria State Government

^e^Senior Alert

^f^Folkhälsomyndigheten

^g^Kolada

### Methodological quality of the QIs

The amount of published information regarding QIs methodological qualities differs per country. For all QI sets the QI purpose, definitions with numerator and denominator, exclusion criteria and risk-adjustment information were publicly available. We found published information on the selection or development of QIs for six countries (U.S., Canada, Australia, Belgium, the Netherlands and partly Sweden). For three countries (Norway, New Zealand and Australia), we received more information on QI development via e-mail contact. All countries involved stakeholders in the development of QIs, however the detailed information was not always available. For Norway, we only know that stakeholders were consulted (e-mail communication), for the Netherlands [79], New Zealand (e-mail communication) and Sweden’s medication-related QIs [94], we also know, which expert groups were included. Regarding QIs from Belgium [80] and most QIs from the U.S. [51], the description of some assessment criteria for the evaluation of QIs, such as relevance or influenceability by RLTC institutions, is available. We were only able to find published results of an expert assessment for the Australian QI set [93] and for six of the Canadian QIs [92].

We found little up-to-date information regarding the validity and reliability of the QIs. Two studies assessed the validity of MDS 2.0 QIs using the same data (209 U.S. RLTC institutions) and validity assessment method, but the results differ depending on applied inclusion criteria and risk-adjustment [25, 39]. Only QIs currently used in Canada apply the same or a very similar measurement as the QIs reported in the study by Jones et al. (2010), showing mostly moderate and in some cases insufficient validity [25]. The U.S. and New Zealand apply no or a different risk-adjustment and use different data collection instruments (i.e., MDS 3.0 and interRAI LTCF respectively). Other studies investigated sensitivity or ability to reflect differences in care between facilities with higher and lower QI scores in multiple [23, 28] or single MDS 2.0 QIs [19, 22, 24, 30–34].

Studies assessing reliability used Kappa statistics. The higher Kappa (range 0–1), the higher the agreement between the two independent raters (interrater reliability) or the higher the consistency of the same rater’s assessment at different timepoints (intrarater reliability). We found interrater reliability results for 17 MDS 2.0-based QIs showing moderate to good Kappa values (0.52–0.89) [39] and on a pressure ulcers QI from the Netherlands (Kappa 0.97) [21]. The reported information on validity and reliability measures per country and per indicator can be found in Additional file 5.

### Methodological assessment of the QI sets with AIRE tool

We assessed the methodological quality of a total of 99 QIs from eight countries using the AIRE instrument. As the information regarding domains 1 and 2 for each QI was mostly applicable to all QIs in a set, we decided to evaluate domain 1 and 2 per country, while in domains 3 and 4 each QI was evaluated separately. Assessment results for each domain per country are reported in Table 4 and the results for each QI can be found in Additional file 3.

**Table 4:** Methodological assessment of the QI sets with AIRE tool

AIRE Domain	US	CA	NZ	AU	BE	NL	NO	SE
**Domain 1: Purpose, relevance and organizational context**								
1.The purpose of the indicator is described clearly	98%	96%	71%	89%	93%	78%	100%	87%
2. The criteria for selecting the topic of the indicator are described in detail								
3.The organizational context of the indicator is described in detail								
4.The quality domain the indicator addresses is described in detail								
5.The health-care process covered by the indicator is described and defined in detail								
**Domain 2: Stakeholder Involvement**								
6. The group developing the indicator includes individuals from relevant professional groups	96%	100%	93%	100%	100%	74%	41%	56%
7. Considering the purpose of the indicator, all relevant stakeholders have been involved at some stage of the development process								
8. The indicator has been formally endorsed								
**Domain 3: Scientific evidence**								
9. Systematic methods were used to search for scientific evidence	0–7%	11–33%	0%	52–56%	0–26%	0–15%	4%	0–7%
10. The indicator is based on recommendations from an evidence-based guideline or studies published in peer-reviewed scientific journals								
11. The supporting evidence has been critically appraised								
**Domain 4: Additional evidence, formulation and usage**								
12. The numerator and denominator are described in detail	44–68%	33–60%	33–52%	52–58%	38–39%	18–32%	31–32%	30–41%
13. The target patient population of the indicator is defined clearly								
14. A strategy for risk adjustment has been considered and described								
15. The indicator measures what it is intended to measure								
16. The indicator measures accurately and consistently								
17. The indicator has sufficient discriminative power								
18. The indicator has been piloted in practice								
19. The efforts needed for data collection have been considered								
20. Specific instructions for presenting and interpreting the indicator results are provided								

The percentages are standardized scores per domain (range 0–100%). A higher score indicates a higher methodological quality. Domain 1 and domain 2 were evaluated by set (i.e., the evaluation was made for the whole set of QIs from the country). In the domains 3 and 4 each QI was evaluated separately and a range is reported. Detailed evaluation is reported in Additional file 3. Item description follows Kieft et al. [96] and Wagner et al. [18]

*Abbreviations US* United States, *CA* Canada, *NZ* New Zealand, *AU* Australia, *BE* Belgium, *NL* Netherlands, *NO* Norway, *SE* Sweden

As presented in Table 4, most QI sets scored well in domain 1 regarding purpose, relevance and organizational context. For New Zealand and the Netherlands, we found little information regarding criteria for selecting the topic of the indicator, which caused a lower rating. The rating in domain 2 shows good ratings for stakeholders’ involvement, except for the Norwegian and Swedish QI sets, where detailed information was not available or scant. Poor performance in domain 3 on scientific evidence has to do with the fact that most countries’ reports stated that a literature search was carried out, but fail to give further details, such as search strategies, reference list of studies based on which the decisions were made, or, if the used studies are referenced, then the quality of the studies was not assessed. In domain 4, the definitions of the QIs and their target groups were consistently well-described and, in most countries, there were also good indications for the display and interpretation of the results. Most frequently lacking was information on validity, discriminative power, testing in practice and the effort required for data collection. Only the Australian indicator set reached high methodological quality, defined as scores of 50% or higher in all four AIRE instrument domains [14].

## Discussion

This review identified 99 publicly reported health-related QIs covering 31 themes in eight countries. The most relevant and up-to-date information was found in grey literature. Peer-reviewed studies with scientific investigations could be found almost only for MDS-based QIs. The themes most covered by the QIs were pressure ulcers, falls, physical restraints and weight loss and often several indicators were used to assess one theme. An Australian review report from 2020, which next to health-related QIs included also QIs related to social well-being and quality of life identified a total of 305 internationally reported QIs for residential aged care (both long-term and short-term) in 11 countries [97]. These results indicate high interest in quality and safety assessment in RLTC in developed countries and a large variety of quality measures. Additionally, a recent article compared the measurement of pressure ulcers with four different approaches in more than 25 countries. Although they found methodological differences in point prevalence measurement that hinder comparison, it shows on the one hand the international interest in comparing QI data and on the other hand the importance of methodological soundness in QI development and measurement [98].

We found limited publicly available up-to-date scientific evidence on the RLTC QIs currently in use, which is consistent with findings of previous reviews [12, 13]. The amount of published information on QIs differs between countries. This could be related to the different stages of development and implementation of RLTC QIs and their public reporting. The U.S. has over 20 years of experience in public reporting of QIs in the RLTC [99], while in Australia, first mandatory measurement results at national level were published in 2019. Most studies reporting on the assessment of QIs were carried out in the U.S. in the first years following the introduction of MDS-based indicators. Since then, the measurement of the U.S. QIs has changed in several cases, which is why some of the evidence is no longer applicable or can only be used to a limited extent. Australia, which was the last country to introduce a nationally publicly reported set of QIs, scored best on the AIRE assessment. It may be because national and international interest in transparency increased over the years. On the other hand, in some countries testing may be still planned or in progress and no results can be reported yet.

All countries published information on their RLTC QIs on websites. While it may be a good solution for publishing guides on QI measurement or results, which change with each reporting period, peer-reviewed journal publications seem better suited for reporting on the development and validation of the QIs. It could improve accessibility of the research for the international public. Moreover, it would strengthen the reporting on scientific evidence (e.g., providing details on the used search methods and critical appraisal of the results). Finally, it would prevent loss of accessibility to the reports due to content transfer or removal, e.g., when the website is no longer maintained, as was the case with information published by Health Quality Ontario cited in this review [92]. The lack of scientific publications might be based on the process of QI development, which is often government-initiated and results in government reports with no further funding for the scientific reporting. Here, a rethinking at government level would be needed to strengthen the scientific approach with peer-reviewed publications.

Publicly reported QIs serve different purposes, which partly drives the way the data is collected and reported. We found little evidence regarding the discriminatory power of QIs meant for providers’ comparisons [11]. This is an important aspect both for quality improvement and for comparing service providers (e.g., through benchmarking) [11, 100–102]. Benchmarking based on QIs which are not able to identify differences of quality of care beyond chance between facilities or regions can lead to wrong or misleading quality assessments, rankings or comparisons. This can in turn result in inappropriate policies or decisions, unfair treatment of the care providers or misguided quality improvement projects [11, 100–102]. Furthermore, while risk-adjustment might not be needed when the goal of the RLTC institutions is to track their own results over time, it is recommended when QIs are used to compare facilities and benchmark them to another or to a set threshold [103].

Some countries, like the U.S. and Canada, use QIs for comparisons between providers, while other mainly aim for quality monitoring and improvement. Both purposes can conceptually lead to better care according to Berwick et al. [104]. On one hand, comparing providers will allow consumers to select better care providers, and might allow regulatory bodies to identify problematic facilities, but it might not fundamentally change the overall performance of the sector. On the other hand, using QIs with the aim to directly improve facilities’ own care processes, and ultimately outcomes, might actually achieve changes in the care quality provided [104]. Ideally, both dynamics would operate stimulatingly for continuous quality improvement of the sector [104]. In practice, using QIs to support quality improvement is difficult and barriers have been identified, such as the lack of organizational and professional skills and capacity in the healthcare sector to use this data for change, or issues with QI measurements [104]. The development, measurement and reporting of QIs depend on their primary purpose. Therefore, countries using or planning to use QIs in RLTC need to coherently construct QIs matching their main purpose [10] and invest in the RLTC sector to fill in deficiencies in capacity linked to QI usage for quality improvement.

### Implications for practice, policy and research

Countries should be encouraged to share more broadly and transparently information on the selection, development, evaluation and reporting of QIs. The AIRE criteria can provide guidance regarding aspects to consider in this process. QIs with satisfying measurement properties increase the possibility for countries, regions or facilities to be using QIs in a useful and informative way and for policy-makers to make informed decisions. Increasing published and accessible information on QI sets would also allow researchers to make international comparisons more easily.

### Limitations

This review has some limitations. First, it is possible that we have not identified all publicly-reported QI sets because of language barriers. Second, only one team member was primarily responsible for initial and full-text screening, therefore relevant information might have been missed. Third, the AIRE assessment is heavily dependent on the available sources of information and the completeness of the documentation for the assessed QIs. Our rating is hence limited by the lack of available information (e.g., it is possible that the information exists but only in a document intended for internal use) or the difficulty of finding published documents (e.g., due to language barriers). For this reason, the AIRE assessment can only reflect the quality of the QIs on the basis of the publicly available data. In this sense, a low rating in some domains does not necessarily mean that the indicators are of low quality. Lastly, the number, types and measurement of internationally reported QIs can change with each following reporting period. For instance, in Australia the nationally reported QI set was extended in July 2021 [41]. However, this did not change our overall quality assessment findings and we did not integrate changes concerning new or changed QIs in our results. A new QI set was also developed and tested in Germany [105–107], but public reporting of the collected data was postponed due to COVID-19 pandemic [108].

## Conclusion

A growing number of countries are using and reporting QI results on a wide range of themes with various purposes and in different ways. Publicly available information on the RLTC QIs was limited for a number of methodological aspects. There is a need for better reporting on the selection and development of QIs, so that these can be used in a useful way to ultimately improve the quality of care provided to the residents.

## Supplementary Information

**Additional file 1.** Search strings used in the databases.

**Additional file 2.** List of consulted websites and organizations.

**Additional file 3.** List of included QIs with type of QI, measurement level and AIRE rating.

**Additional file 4.** List of articles excluded after full-text screening.

**Additional file 5.** Reported measurement properties on validity and reliability per QI.

## Data Availability

Data sharing is not applicable to this article as no datasets were generated or analysed during the study.

## Abbreviations

ADL: Activities of Daily Living
AIRE: Appraisal of Indicators through Research and Evaluation
CMS: Centers for Medicare & Medicaid Servies
LTCF: Long-term care facilitiy
MDS: Minimum Data Set
MeSH: Medical Subject Heading
QI: Quality indicator
RAI: Resident Assessment Instrument
RLTC: Residential long-term care
U.S.: United States
UTI: Urinary tract infection
