# Supplementary material for: Evidence for publicly reported quality indicators in residential long-term care: a systematic review
Source: BMC Health Serv Res. 2022 Nov 24;22:1408. doi: 10.1186/s12913-022-08804-7 (PMC9686098; doi:10.1186/s12913-022-08804-7)
Supplement: Supplementary file 1 — Additional file 1. Search strings used in the databases. [file 12913_2022_8804_MOESM1_ESM.docx]

**Additional file 1: Search strings used in the databases.**

***PubMed***

("nursing homes"[MeSH] OR "long term care"[MeSH] OR "homes for the aged"[MeSH] OR nursing home[tiab] OR nursing homes[tiab] long-term care[tiab] OR home for the aged[tiab] OR homes for the aged[tiab] OR care home*[tiab] OR residential care[tiab] OR residential aged care[tiab] OR nursing facilit*[tiab] OR aged care facilit*[tiab])

AND

("Quality Indicators, Health Care"[Mesh] OR "Quality Assurance, Health Care/methods"[Mesh] OR "Quality Assurance, Health Care/statistics and numerical data"[Mesh] OR "Quality Assurance, Health Care/standards"[Mesh] OR "Quality of Health Care/Standards"[Mesh] OR “Quality of Health Care/statistics and numerical data” [Mesh] OR "Quality Improvement"[Mesh] OR “Patient Outcome Assessment” [Mesh] OR quality indicator*[tiab] OR quality assessment*[tiab] OR quality measure*[tiab] OR performance assessment*[tiab] OR performance indicator* [tiab] OR performance measure*[tiab] OR care quality[tiab] OR quality of care[tiab] OR quality of health care[tiab] OR quality of healthcare[tiab] OR quality of nursing care[tiab] OR quality improvement* [tiab] OR resident outcome*[tiab] OR patient outcome*[tiab] OR "Mandatory Reporting"[Mesh] OR "Information Dissemination"[Mesh] OR "Benchmarking"[Mesh] OR mandatory report*[tiab] OR public report*[tiab] OR report card*[tiab] OR benchmarking [tiab] OR information dissemination [tiab])

***CINAHL***
(MH "Nursing Homes+" OR MH "Long Term Care" OR TI ( "nursing home*" OR "long-term care" OR "home* for the aged" OR "care home*" OR "residential care" OR "residential aged care" OR "nursing facilit*" OR "aged care facilit*" ) OR AB ( "nursing home" OR "nursing homes" OR "long-term care" OR "home for the aged" OR "homes for the aged" OR "care home*" OR "residential care" OR "residential aged care" OR "nursing facilit*" OR "aged care facilit*" ))

AND
(MH "Clinical Indicators" OR MH "Quality Assurance/ST/MT/SN" OR MH "Quality of Health Care/ST/SN" OR MH "Quality Improvement" OR MH "Outcome Assessment" OR TI ( "quality indicator*" OR "quality assessment*" OR "quality measure*" OR "performance assessment*" OR "performance indicator*" OR "performance measure*" OR "care quality" OR "quality of care" OR "quality of health care" OR "quality of healthcare" OR "quality of nursing care" OR "quality improvement*" OR "resident outcome*" OR "patient outcome*" ) OR AB ( "quality indicator*" OR "quality assessment*" OR "quality measure*" OR "performance assessment*" OR "performance indicator*" OR "performance measure*" OR "care quality" OR "quality of care" OR "quality of health care" OR "quality of healthcare" OR "quality of nursing care" OR "quality improvement*" OR "resident outcome*" OR "patient outcome*") OR TI ("mandatory report*" OR "public report*" OR "report card*" OR "benchmarking" OR "information dissemination" ) OR AB ("mandatory report*" OR "public report*" OR "report card*" OR "benchmarking" OR "information dissemination" ) OR MH "Mandatory Reporting" OR MH "Benchmarking")

***EMBASE***

nursing home/ or long term care/ or home for the aged/ or nursing home.ab,ti. or nursing homes.ab,ti. or long term care.ab,ti. or home for the aged.ab,ti. or homes for the aged.ab,ti. or care home*.ab,ti. or residential care/ or residential care.ab,ti. or residential aged care.ab,ti. or nursing facilit*.ab,ti. or aged care facilit*.ab,ti.

and

clinical indicator/ or quality control/ or total quality management/ or quality indicator*.ab,ti. or quality assessment*.ab,ti. or quality measure*.ab,ti. or performance assessment*.ab,ti. or performance indicator*.ab,ti. or performance measure*.ab,ti. or care quality.ab,ti. or quality of care.ab,ti. or quality of health care.ab,ti. or quality of healthcare.ab,ti. or quality of nursing care.ab,ti. or quality improvement*.ab,ti. or resident outcome*.ab,ti. or patient outcome*.ab,ti. or mandatory reporting/ or information dissemination/ or benchmarking/ or mandatory report*.ab,ti. or public report*.ab,ti. or report card*.ab,ti. or benchmarking.ab,ti. or information dissemination.ab,ti.
