# Supplementary material for: Evidence for publicly reported quality indicators in residential long-term care: a systematic review
Source: BMC Health Serv Res. 2022 Nov 24;22:1408. doi: 10.1186/s12913-022-08804-7 (PMC9686098; doi:10.1186/s12913-022-08804-7)
Supplement: Supplementary file 2 — Additional file 2. List of consulted websites and organizations. [file 12913_2022_8804_MOESM2_ESM.docx]

**Additional file 2: List of consulted websites and contacted organizations.**

| **Organisation / Initiative** | **Country** | **Link** | **Relevant information on QIs** | **E-mail for further information** |
| --- | --- | --- | --- | --- |
| interRAI | International | <https://www.interrai.org> |  |  |
| LPZ | International | <https://ch.lpz-um.eu/de/Home/Publications> |  |  |
| Organisation for Economic Co-operation and Development (OECD): | International | <http://www.oecd.org/health/long-term-care.htm> |  |  |
| Abt Associates Inc. | U.S. | <https://www.abtassociates.com> |  |  |
| Agency for Healthcare Research and Quality (AHRQ) | U.S. | <https://www.ahrq.gov> |  |  |
| Centers for Medicare & Medicaid Services (CMS) | U.S. | <https://www.cms.gov/Medicare/Quality-Initiatives-Patient-Assessment-Instruments/NursingHomeQualityInits/NHQIQualityMeasures> | x |  |
| Medicare.gov (managed by CMS) | U.S. | <https://www.medicare.gov/care-compare/> | x |  |
| National Quality Forum (NQF) | U.S. | <https://www.qualityforum.org/Measuring_Performance/Consensus_Development_Process/CSAC_Decision.aspx> | x |  |
| Alberta Health Services | Canada | <https://www.albertahealthservices.ca/about/Page12954.aspx> | x |  |
| Canadian Institute for Health Information (CIHI) | Canada | <https://www.cihi.ca/en/access-data-and-reports/indicators>  <https://yourhealthsystem.cihi.ca> | x | x |
| Health Quality Ontario | Canada | <https://www.hqontario.ca/System-Performance/Measuring-System-Performance/Indicator-Library> | x |  |
| interRAI New Zealand | New Zealand | <https://www.interrai.co.nz/data-and-reporting/quality-indicators> | x | x |
| Australian Government Department of Health | Australia | <https://www.health.gov.au/initiatives-and-programs/national-aged-care-mandatory-quality-indicator-program>  <https://agedcare.health.gov.au> | x | x |
| Australian Institute of Health and Welfare (AIHW) GEN Aged care data | Australia | <https://www.gen-agedcaredata.gov.au/Topics/Quality-in-aged-care> | x |  |
| State of Victoria, Department of Health & Human Services | Australia | <https://www.health.vic.gov.au/residential-aged-care/quality-indicators-in-public-sector-residential-aged-care-services> | x | x |
| Vlaams Instituut voor Kwaliteit van Zorg | Belgium | <https://www.zorgkwaliteit.be/woonzorgcentra> | x | x |
| ActiZ (Nursing Homes Association) | Netherlands | <https://www.actiz.nl/homepage> |  | x |
| National Institute for Public Health and the Environment | Netherlands | <https://www.rivm.nl/en> |  |  |
| Volksgezondheidenzorg.info | Netherlands | <https://www.volksgezondheidenzorg.info/prestatie-indicatoren-voor-gezondheidszorg/leven-met-een-ziekte-beperking#node-nosocomiale-decubitus-instellingen-voor-langdurige-zorg> |  |  |
| Zorginstituut Nederland | Netherlands | <https://www.zorginzicht.nl/openbare-data/open-data-verpleeghuiszorg> | x |  |
| Zorg voor Beter (ActiZ, Zorgthuisnl, Verpleegkundigen &Verzorgenden Nederland and Verenso) | Netherlands | <https://www.zorgvoorbeter.nl/nieuws/indicatoren-verpleeghuiszorg-2019> |  |  |
| Helsedirektoratet | Norway | <https://www.helsedirektoratet.no> / <https://www.helsedirektoratet.no/statistikk> | x | x |
| Folkhälsomyndigheten | Sweden | <https://www.folkhalsomyndigheten.se/halt/> | x |  |
| Rådet för främjande av kommunala analyser (RKA): Kolada | Sweden | <https://www.kolada.se/verktyg/jamforaren/?_p=jamforelse&focus=16680&tab_id=84176> | x | x |
| Senior alert | Sweden | <https://www.senioralert.se> /  <https://plus.rjl.se/infopage.jsf?nodeId=40605> | x | x |
| Socialstyrelsen | Sweden | <https://www.socialstyrelsen.se/statistik-och-data/oppna-jamforelser/socialtjanst/aldreomsorg/> | x | x |
| Bundesarbeitsgemeinschaft der Freien Wohlfahrtspflege | Germany | <https://www.bagfw.de/qualitaet/qualitaetsindikatoren-in-der-stationaeren-pflege> |  |  |
| Geschäftsstelle Qualitätsausschuss Pflege | Germany | <https://www.gs-qsa-pflege.de/dokumente-zum-download> |  |  |
| Finnish Institute for Health and Welfare | Finland | <https://thl.fi/en/web/thlfi-en> |  |  |
| Sotkanet.fi (statistical information on welfare and health in Finland) | Finland | <https://sotkanet.fi/sotkanet/en/haku?g=600> |  |  |
| Care Quality Commission | United Kingdom | <https://www.cqc.org.uk> |  |  |
| Healthcare Improvement Scotland | United Kingdom | <http://www.healthcareimprovementscotland.org> |  |  |
| Health Information and Quality Authority (HIQA) | Ireland | <https://www.hiqa.ie> |  |  |
| ANAP (appui santé et médico-social): | France | <https://www.anap.fr/ressources/publications/detail/actualites/tableau-de-bord-de-la-performance-dans-le-secteur-medico-social-premiers-enseignements/> |  |  |
| Anesm (Agence nationale de l’évaluation et de la quailté des établissements et services sociaux et médico-sociaux) | France | <https://www.has-sante.fr/upload/docs/application/pdf/2018-03/anesm_evaluation_interne_web3.pdf> |  |  |
| Commission des affaires sociales | France | <http://www.assemblee-nationale.fr/15/pdf/rap-info/i1214.pdf> |  |  |
| Observatori del Sistema de Salut de Catalunya | Spain | <http://observatorisalut.gencat.cat/ca/central_de_resultats/> |  |  |
| Statistics Iceland | Iceland | <https://www.statice.is/> |  |  |
| Embætti landlæknis (Directorate of Health) | Iceland | <https://www.landlaeknir.is/gaedi-og-eftirlit/heilbrigdisthjonusta/gaedavisar/rai-gaedavisar/> |  |  |
