# Supplementary material for: Evidence for publicly reported quality indicators in residential long-term care: a systematic review
Source: BMC Health Serv Res. 2022 Nov 24;22:1408. doi: 10.1186/s12913-022-08804-7 (PMC9686098; doi:10.1186/s12913-022-08804-7)
Supplement: Supplementary file 4 — Additional file 4. List of articles excluded after full-text screening. [file 12913_2022_8804_MOESM4_ESM.docx]

**Additional file 4:** **List of articles excluded after full-text screening.**

**No or not relevant information on the measurement, development or reporting of resident health-related quality indicators.**

1. Alexander GL, Madsen RW, Miller E, Wise K. Innovations in Quality Improvement in Long-term Care. A National Report of Nursing Home Information Technology Adoption and Quality Measures. Journal of Nursing Care Quality. 2016;31(3):201-6.
2. Arling G, Lewis T, Kane RL, Mueller C, Flood S. Improving quality assessment through multilevel modeling: the case of nursing home compare. Health Serv Res. 2007;42(3 Pt 1):1177-99.
3. Aud MA, Rantz MJ, Zwygart-Stauffacher M, Manion P. Developing a residential care facility version of the observable indicators of Nursing Home Care Quality Instrument. J Nurs Care Qual. 2004;19(1):48-57.
4. Berlowitz DR, Brandeis GH, Anderson JJ, Ash AS, Kader B, Morris JN, et al. Evaluation of a risk-adjustment model for pressure ulcer development using the minimum data set. Journal of the American Geriatrics Society. 2001;49(7):872-6.
5. Berlowitz DR, Brandeis GH, Morris JN, Ash AS, Anderson JJ, Kader B, et al. Deriving a risk-adjustment model for pressure ulcer development using the minimum data set. Journal of the American Geriatrics Society. 2001;49(7):866-71.
6. Berlowitz DR, Christiansen CL, Brandeis GH, Ash AS, Kader B, Morris JN, et al. Profiling nursing homes using Bayesian hierarchical modeling. Journal of the American Geriatrics Society. 2002;50(6):1126-30.
7. Castle NG. Outcomes measurement and quality improvement in long-term care. J Healthc Qual. 1999;21(3):21-5.
8. Castle NG, Lowe TJ. Report cards and nursing homes. Gerontologist. 2005;45(1):48-67.
9. Cotter AJE, Salvage AV, Meyer JE, Bridges J. Measuring outcomes of long-term care for older people. Reviews in Clinical Gerontology. 1998;8(3):257-68.
10. Davila H, Shippee TP, Park YS, Brauner D, Werner RM, Konetzka RT. Inside the Black Box of Improving on Nursing Home Quality Measures. Medical Care Research and Review. 2020.
11. de Stampa M, Cerase V, Bagaragaza E, Lys E, Alitta Q, Gammelin C, et al. Implementation of a Standardized Comprehensive Assessment Tool in France: A Case Using the InterRAI Instruments. International Journal of Integrated Care (IJIC). 2018;18(2):1-8.
12. Delaney CM, Rafalson L, Fiedler RC, Hernick JI. Quality indicator survey versus traditional survey in New York State: a comparison of results from annual nursing home surveys. Journal of Aging & Social Policy. 2018;30(2):127-40.
13. Du Moulin MF, van Haastregt JC, Hamers JP. Monitoring quality of care in nursing homes and making information available for the general public: state of the art. Patient Education & Counseling. 2010;78(3):288-96.
14. Faust R. Indicators for the sensible judgement of care-sensitivity quality in nursing home compare -- chances of RAI MDS-based quality indicators for quality measurement of nursing in Germany. PR-Internet fur die Pflege. 2004;6(4):222-30.
15. Grant NK, Reimer M, Bannatyne J. Indicators of quality in long-term care facilities. Int J Nurs Stud. 1996;33(5):469-78.
16. Groenewoud AF, van Exel NJA, Berg M, Huijsman R. Building quality report cards for geriatric care in The Netherlands: using Concept Mapping to identify the appropriate 'building blocks' from the consumer's perspective. Gerontologist. 2008;48(1):79-92.
17. Gustafson DH, Sainfort FC, Konigsveld RV, Zimmerman DR. The quality assessment index (QAI) for measuring nursing home quality. Health Services Research. 1990;25(1 I):97-127.
18. Herr A, Nguyen T-V, Schmitz H. Public reporting and the quality of care of German nursing homes. Health Policy. 2016;120(10):1162-70.
19. Hutchinson AM, Draper K, Sales AE. Public reporting of nursing home quality of care: lessons from the United States experience for Canadian policy discussion. Healthcare Policy. 2009;5(2):87-105.
20. Hyang Yuol L, Juh Hyun S. Public Reporting on the Quality Ratings of Nursing Homes in the Republic of Korea. Journal of Korean Academy of Nursing. 2019;49(2):161-70.
21. Ibrahim JE, Chadwick L, MacPhail A, McAuliffe L, Koch S, Wells Y. Use of Quality Indicators in Nursing Homes in Victoria, Australia: A Cross-Sectional Descriptive Survey. Journal of Aging & Health. 2011;26(5):824-40.
22. Inacio MC, Lang C, Caughey GE, Bray SCE, Harrison SL, Whitehead C, et al. The Registry of Senior Australians outcome monitoring system: quality and safety indicators for residential aged care. Int J Qual Health Care. 2020;32(8):502-10.
23. Ivers NM, Taljaard M, Giannakeas V, Reis C, Williams E, Bronskil S. Public reporting of antipsychotic prescribing in nursing homes: population-based interrupted time series analyses. BMJ Quality & Safety. 2019;28(2):121-31.
24. Jensdottir AB, Rantz M, Hjaltadottir I, Gudmundsdottir H, Rook M, Grando V. International comparison of quality indicators in United States, Icelandic and Canadian nursing facilities. Int Nurs Rev. 2003;50(2):79-84.
25. Lin MK, Kramer AM. The Quality Indicator Survey: Background, Implementation, and Widespread Change. Journal of Aging and Social Policy. 2013;25(1):10-29.
26. Mashouri P, Taati B, Quirt H, Iaboni A. Quality Indicators as Predictors of Future Inspection Performance in Ontario Nursing Homes. J Am Med Dir Assoc. 2020;21(6):793-8.e1.
27. Möller J, Panhorst H, Zieres G. Public reporting on professional nursing home care: demand and reality. Gesundheitsökonomie & Qualitätsmanagement. 2010;15(4):185-91.
28. Mor V. Defining and measuring quality outcomes in long-term care. J Am Med Dir Assoc. 2006;7(8):532-8; discussion 8-40.
29. Mor V, Angelelli J, Gifford D, Morris J, Moore T. Benchmarking and quality in residential and nursing homes: lessons from the US. Int J Geriatr Psychiatry. 2003;18(3):258-66.
30. Mor V, Berg K, Angelelli J, Gifford D, Morris J, Moore T. The quality of quality measurement in U.S. nursing homes. Gerontologist. 2003;43(SPEC. ISS. 2):37-46.
31. Mor V, Intrator O, Unruh MA, Cai S. Temporal and Geographic variation in the validity and internal consistency of the Nursing Home Resident Assessment Minimum Data Set 2.0. BMC Health Serv Res. 2011;11:78.
32. Morris JN, Hawes C, Fries BE, Phillips CD, Mor V, Katz S, et al. Designing the national resident assessment instrument for nursing homes. Gerontologist. 1990;30(3):293-307.
33. Mukamel DB. Risk-adjusted outcome measures and quality of care in nursing homes. Medical care. 1997;35(4):367-85.
34. Mukamel DB, Brower CA. The influence of risk adjustment methods on conclusions about quality of care in nursing homes based on outcome measures. Gerontologist. 1998;38(6):695-703.
35. Mukamel DB, Glance LG, Li Y, Weimer DL, Spector WD, Zinn JS, et al. Does risk adjustment of the CMS quality measures for nursing homes matter? Medical Care. 2008;46(5):532-41.
36. Ning JZ, Wan TTH. The measurement of nursing home quality: Multilevel confirmatory factor analysis of panel data. Journal of Medical Systems. 2005;29(4):401-11.
37. Norton PG, Murray M, Doupe MB, Cummings GG, Poss JW, Squires JE, et al. Facility versus unit level reporting of quality indicators in nursing homes when performance monitoring is the goal. BMJ Open. 2014;4(2):e004488.
38. Oliveira WI, Hernandez PJ, Sousa Kde M, Piuvezam G, Gama ZA. [Semantic and conceptual equivalence of the observable indicatorsof Nursing Home Care Quality Instrument]. Cien Saude Colet. 2016;21(7):2243-56.
39. Onder G, Carpenter I, Finne-Soveri H, Gindin J, Frijters D, Henrard JC, et al. Assessment of nursing home residents in Europe: the Services and Health for Elderly in Long TERm care (SHELTER) study. BMC Health Serv Res. 2012;12:5.
40. Phillips CD, Zimmerman D, Bernabei R, Jonsson PV. Using the Resident Assessment Instrument for quality enhancement in nursing homes. Age Ageing. 1997;26 Suppl 2:77-81.
41. Poldrugovac M, Klazinga NS, Kringos DS, Padget M, Schoonhoven L, Thompson ND. International comparison of pressure ulcer measures in long-term care facilities: Assessing the methodological robustness of 4 approaches to point prevalence measurement. Journal of Tissue Viability. 2021.
42. Przylog A, Stroka MA, Engel S, Linder R. Do nursing homes with higher quality ratings provide a better quality of care? : Empirical study based on administrative data. Zeitschrift fur Gerontologie und Geriatrie. 2016;49(4):308-16.
43. Rantz M, Jensdóttir AB, Hjaltadóttir I, Gudmundsdóttir H, Gudjónsdóttir JS, Brunton B, et al. International field test results of the Observable Indicators of Nursing Home Care quality instrument. International Nursing Review. 2002;49(4):234-42.
44. Rantz MJ, Mehr DR, Petroski GF, Madsen RW, Popejoy LL, Hicks LL, et al. Initial field testing of an instrument to measure: observable indicators of nursing home care quality. Journal of Nursing Care Quality. 2000;14(3):1-12.
45. Rivera-Hernandez M, Matos-Moreno A, Ferdows NB, Kumar A. Posthospital Nursing Home Utilization and Quality Indicators Among Medicare Beneficiaries in Puerto Rico: Comparison With the United States. Journal of the American Medical Directors Association. 2021;22(3):712.
46. Rosen AK, Berlowitz DR, Anderson JJ, Ash AS, Kazis LE, Moskowitz MA. Functional status outcomes for assessment of quality in long-term care. Int J Qual Health Care. 1999;11(1):37-46.
47. Ryan J, Stone RI, Raynor CR. Using large data sets in long-term care to measure and improve quality. Nurs Outlook. 2004;52(1):38-44.
48. Sales A, O'Rourke HM, Draper K, Teare GF, Maxwell C. Prioritizing information for quality improvement using resident assessment instrument data: experiences in one Canadian province. Healthcare Policy. 2011;6(3):55-67.
49. Sgadari A, Morris JN, Fries BE, Ljunggren G, Jonsson PV, Dupaquier JN, et al. Efforts to establish the reliability of the Resident Assessment Instrument. Age and Ageing. 1997;26(SUPPL. 2):27-30.
50. Shwartz M, Burgess J, Berlowitz D. Benefit-of-the-doubt approaches for calculating a composite measure of quality. Health Services & Outcomes Research Methodology. 2009;9(4):234-51.
51. Shwartz M, Peköz EA, Christiansen CL, Burgess JF, Jr., Berlowitz D, Shwartz M, et al. Shrinkage estimators for a composite measure of quality conceptualized as a formative construct. Health Services Research. 2013;48(1):271-89.
52. Sunderkamp S, Weiss C, Rothgang H. [Analysis of public quality reports for home care and long-term care with respect to their usefulness for the customer]. Pflege. 2014;27(5):325-36.
53. Towers A-M, Palmer S, Smith N, Collins G, Allan S. A cross-sectional study exploring the relationship between regulator quality ratings and care home residents' quality of life in England. Health & Quality of Life Outcomes. 2019;17(1):1-11.
54. Trigg L, Kumpunen S, Holder J, Maarse H, SolÉ JuvÉS M, Gil J. Information and choice of residential care provider for older people: a comparative study in England, the Netherlands and Spain. Ageing & Society. 2018;38(6):1121-47.
55. Wagner LM, McDonald SM, Castle NG. Joint Commission Accreditation and Quality Measures in U.S. Nursing Homes. Policy, Politics & Nursing Practice. 2012;13(1):8-16.
56. Winter JD, Kerns JW, Winter KM, Richards A, Sabo RT. Unreported Antipsychotic Use Increasing in Nursing Homes: The Impact of Quality-Measure Exclusions on the Percentage of Long-Stay Residents Who Got an Antipsychotic Medication Quality-Measure. American Journal of Geriatric Psychiatry. 2020.
57. Winters S, Strating MH, Klazinga NS, Kool RB, Huijsman R. Determining the interviewer effect on CQ Index outcomes: a multilevel approach. BMC medical research methodology. 2010;10:75.
58. Winters-Van Der Meer S, Kool RB, Klazinga NS, Huijsman R. Are the Dutch long-term care organizations getting better? A trend study of quality indicators between 2007 and 2009 and the patterns of regional influences on performance. Int J Qual Health Care. 2013;25(5):505-14.
59. Xu D, Kane RL, Shippee T, Lewis TM. Identifying Consistent and Coherent Dimensions of Nursing Home Quality: Exploratory Factor Analysis of Quality Indicators. J Am Geriatr Soc. 2016;64(12):e259-e64.
60. Zhang NJ, Paek SC, Wan TT, Zhang NJ, Paek SC, Wan TTH. Reliability estimates of clinical measures between Minimum Data Set and Online Survey Certification and Reporting data of US nursing homes. Medical Care. 2009;47(4):492-5.

**Not currently publicly reported indicators (different indicator, data collection method or operationalization) or no information on public reporting provided.**

1. Bates-Jensen BM, Alessi CA, Cadogan M, Levy-Storms L, Jorge J, Yoshii J, et al. The Minimum Data Set bedfast quality indicator: differences among nursing homes. Nursing Research. 2004;53(4):260-72.
2. Bates-Jensen BM, Cadogan M, Jorge J, Schnelle JF. Standardized quality-assessment system to evaluate pressure ulcer care in the nursing home. Journal of the American Geriatrics Society. 2003;51(9):1195-202.
3. Bates-Jensen BM, Simmons SF, Schnelle JF, Alessi C. Evaluating the accuracy of minimum data set bed-mobility ratings against independent performance assessments: systematic error and directions for improvement. Gerontologist. 2005;45(6):731-8.
4. Bell JS, Taipale HT, Soini H, Pitkala KH. Prognostic value of the quality indicator "concurrent use of three or more psychotropic drugs" among residents of long-term-care facilities. Eur J Clin Pharmacol. 2009;65(11):1163-4; author reply 1.
5. Boerlage A, Masman A, Tibboel D, Baar F, Van Dijk M. Is pain measurement a feasible performance indicator for Dutch nursing homes? A cross-sectional approach. European Journal of Pain. 2009;13(SUPPL. 1):S45.
6. Boerlage AA, Masman AD, Hagoort J, Tibboel D, Baar FPM, van Dijk M. Is Pain Assessment Feasible as a Performance Indicator for Dutch Nursing Homes? A Cross-Sectional Approach. Pain Management Nursing. 2013;14(1):36-40.
7. Carryer J, Weststrate J, Yeung P, Rodgers V, Towers A, Jones M. Prevalence of key care indicators of pressure injuries, incontinence, malnutrition, and falls among older adults living in nursing homes in New Zealand. Research in Nursing & Health. 2017;40(6):555-63.
8. Castle NG. Providing outcomes information to nursing homes: can it improve quality of care? Gerontologist. 2003;43(4):483-92.
9. Castle NG, Degenholtz H, Engberg J. State variability in indicators of quality of care in nursing facilities. J Gerontol A Biol Sci Med Sci. 2005;60(9):1173-9.
10. Chung J. Development and application of nursing service quality indicators in nursing homes. Taehan Kanho Hakhoe chi. 2007;37(3):401-13.
11. Courtney M, O'Reilly MT, Edwards H, Hassall S. Development of a systematic approach to assessing quality within Australian residential aged care facilities: the Clinical Care Indicators Tool with med QIs. Aust Health Rev. 2007;31(4):582-91.
12. Courtney M, OÂ¿Reilly M, Edwards H, Hassall S. Content validity of the ResCareQA: An Australian residential care quality assessment based on resident outcomes. Australian Journal of Advanced Nursing. 2011;28(3):37-46.
13. Denny DS, Christian V, Nordan VN, Therriault MF. Comparative data analysis using collaborative skilled nursing/long-term care indicator assessment. J Healthc Qual. 1998;20(4):12-20; quiz 1, 52.
14. Domhoff D, Seibert K, Stiefler S, Wolf-Ostermann K, Peschke D. Differences in nursing home admission between functionally defined populations in Germany and the association with quality of health care. BMC Health Serv Res. 2021;21(1):190.
15. García-Altés A, Subirana-Casacuberta M, Llorens D, Bullich I, Brugués A, Teixidor M, et al. The experience of Catalonia measuring nurse-sensitive indicators: Trends study 2012-2018. J Nurs Manag. 2021.
16. Heiser D. Depression Identification in the Long-Term Care Setting. Clinical Gerontologist. 2004;27(4):3-18.
17. Hjaltadottir I, Ekwall AK, Nyberg P, Hallberg IR. Quality of care in Icelandic nursing homes measured with Minimum Data Set quality indicators: retrospective analysis of nursing home data over 7 years. Int J Nurs Stud. 2012;49(11):1342-53.
18. Hjelmar U, Bhatti Y, Petersen OH, Rostgaard T, Vrangbaek K. Public/private ownership and quality of care: Evidence from Danish nursing homes. Soc Sci Med. 2018;216:41-9.
19. Igarashi A, Eltaybani S, Takaoka M, Noguchi-Watanabe M, Yamamoto-Mitani N. Quality Assurance in Long-Term Care and Development of Quality Indicators in Japan. Gerontology and Geriatric Medicine. 2020;6.
20. Li Y, Cai X, Glance LG, Spector WD, Mukamel DB, Li Y, et al. National release of the nursing home quality report cards: implications of statistical methodology for risk adjustment. Health Services Research. 2009;44(1):79-102.
21. Li Y, Schnelle J, Spector WD, Glance LG, Mukamel DB. The "Nursing Home Compare" measure of urinary/fecal incontinence: cross-sectional variation, stability over time, and the impact of case mix. Health Serv Res. 2010;45(1):79-97.
22. Liu C, Feng Z, Mor V. Case-mix and quality indicators in Chinese elder care homes: are there differences between government-owned and private-sector facilities? J Am Geriatr Soc. 2014;62(2):371-7.
23. Mueller C, Karon SL. ANA nurse sensitive quality indicators for long-term care facilities. J Nurs Care Qual. 2004;19(1):39-47.
24. Mukamel DB, Watson NM, Meng H, Spector WD. Development of a risk-adjusted urinary incontinence outcome measure of quality for nursing homes. Medical Care. 2003;41(4):467-78.
25. Mylotte JM. Risk adjustment for benchmarking nursing home infection surveillance data: A narrative review. American Journal of Infection Control. 2021;49(3):366-74.
26. Nakrem S, Vinsnes AG, Harkless GE, Paulsen B, Seim A. Nursing sensitive quality indicators for nursing home care: international review of literature, policy and practice. International Journal of Nursing Studies. 2009;46(6):848-57.
27. O'Reilly M, Courtney M, Edwards H. How is quality being monitored in Australian residential aged care facilities? A narrative review. Int J Qual Health Care. 2007;19(3):177-82.
28. O'Reilly M, Courtney M, Edwards H, Hassall S. Clinical outcomes in residential care: setting benchmarks for quality. Australas J Ageing. 2011;30(2):63-9.
29. Palm R, Schmidt S, Galatsch M, Hasselhorn H-M, Muller BH. Quality indicators in practice - results of expert interviews of the 3Q-study. Pflegewissenschaft. 2011;13(4):213-22.
30. Phillips CJ. Developing a method of assessing quality of care in nursing homes, using key indicators and population norms. Journal of Aging and Health. 1991;3(3):407-22.
31. Pileggi C, Manuti B, Costantino R, Bianco A, Nobile CGA, Pavia M. Quality of care in one Italian nursing home measured by ACOVE process indicators. PLoS ONE. 2014;9(3):e93064.
32. Porell F, Caro FG. Facility-level outcome performance measures for nursing homes. Gerontologist. 1998;38(6):665-83.
33. Rahman M, Grabowski DC, Mor V, Norton EC. Is a Skilled Nursing Facility's Rehospitalization Rate a Valid Quality Measure? Health Serv Res. 2016;51(6):2158-75.
34. Reichert AR, Stroka MA. Nursing home prices and quality of care - Evidence from administrative data. Health Economics. 2018;27(1):129-40.
35. Rolland Y, Mathieu C, Piau C, Cayla F, Bouget C, Vellas B, et al. Improving the Quality of Care of Long-Stay Nursing Home Residents in France. Journal of the American Geriatrics Society. 2016;64(1):193-9.
36. Saliba D, Schnelle JF. Indicators of the quality of nursing home residential care. J Am Geriatr Soc. 2002;50(8):1421-30.
37. Sandoval Garrido FA, Tamiya N, Kashiwagi M, Miyata S, Okochi J, Moriyama Y, et al. Relationship between structural characteristics and outcome quality indicators at health care facilities for the elderly requiring long-term care in Japan from a nationwide survey. Geriatr Gerontol Int. 2014;14(2):301-8.
38. Sanubol M. Reliability of six outcomes from the Nursing Outcomes Classification (NOC) and the relationships of organizational, patient and nurse characteristics to these outcomes: University of Iowa; 2005.
39. Schaal T, Schönfelder T, Klewer J, Kugler J. Quality of care in German nursing homes. HeilberufeSCIENCE. 2015;6(1):3-9.
40. Schnelle JF, Wood S, Schnelle ER, Simmons SF. Measurement sensitivity and the Minimum Data Set depression quality indicator. Gerontologist. 2001;41(3):401-5.
41. Shin JH. Nursing Staff Characteristics on Resident Outcomes in Nursing Homes. J Nurs Res. 2019;27(1):1-9.
42. Simmons SF, Cadogan MP, Cabrera GR, Al-Samarrai NR, Jorge JS, Levy-Storms L, et al. The minimum data set depression quality indicator: does it reflect differences in care processes? Gerontologist. 2004;44(4):554-64.
43. Simon M, Pereira O, Hulscher M, Schouten J, Thilly N, Pulcini C. Quantity Metrics and Proxy Indicators to Estimate the Volume and Appropriateness of Antibiotics Prescribed in French Nursing Homes: A Cross-sectional Observational Study Based on 2018 Reimbursement Data. Clin Infect Dis. 2021;72(10):e493-e500.
44. Simon M, Schmidt SG, Schwab CG, Hasselhorn HM, Bartholomeyzcik S. [Determining the quality of long-term care. A comparative analysis of transparency criteria, resident-related indicators, and staff assessment]. Bundesgesundheitsblatt Gesundheitsforschung Gesundheitsschutz. 2013;56(8):1088-97.
45. Sluggett JK, Bell JS, Lang C, Corlis M, Whitehead C, Wesselingh SL, et al. Residential medication management reviews in Australian residential aged care facilities. Malden, Massachusetts: Wiley-Blackwell; 2021. p. 432-3.
46. Xu H, Bowblis JR, Li Y, Caprio TV, Intrator O. Construction and Validation of Risk-adjusted Rates of Emergency Department Visits for Long-stay Nursing Home Residents. Medical Care. 2020;58(2):174-82.
47. Yamamoto-Mitani N, Okamoto Y, Tsujimura M, Kanagawa K, Masaki H, Suzuki M, et al. An examination for developing the quality indicators of home healthcare nursing for older adults: a national self-evaluation survey of nurses working at home care nursing stations. Journal of Japan Academy of Nursing Science. 2008;28(2):37-45.
48. Zisselman MH, Warren RS, Cutillo-Schmitter T, Denman SJ. Challenging the quality of the quality indicator, 'depression without treatment'. Journal of the American Medical Directors Association. 2002;3(2):41-5.

**Not a study (e.g., editorials) or not a primary study**

1. Arling G, Kane RL, Lewis T, Mueller C. Future development of nursing home quality indicators. Gerontologist. 2005;45(2):147-56.
2. Burke RE, Werner RM. Quality measurement and nursing homes: measuring what matters. BMJ Qual Saf. 2019;28(7):520-3.
3. Cefalu C. Nursing Home Quality Measures: Do They Accurately Reflect Quality? Annals of Long Term Care. 2011;19(9):33-40.
4. Du Moulin MF, van Haastregt JC, Hamers JP. Monitoring quality of care in nursing homes and making information available for the general public: state of the art. Patient Education & Counseling. 2010;78(3):288-96.
5. Fukui C, Igarashi A, Noguchi-Watanabe M, Sakka M, Kitamura S, Inagaki A, et al. Development of quality indicators for evaluating the quality of long-term care. Geriatrics and Gerontology International. 2021;21(4):370-1.
6. Hutchinson AM, Milke DL, Maisey S, Johnson C, Squires JE, Teare G, et al. The Resident Assessment Instrument-Minimum Data Set 2.0 quality indicators: a systematic review. BMC health services research. 2010;10:166.
7. McCormack B, McKenna H. Challenges to quality monitoring systems in care homes. Quality in Health Care. 2001;10(4):200-1.
8. Noro A, Björkgren M, Finne-Soveri H. Using quality indicators for benchmarking. Sairaanhoitaja. 2002;75(6-7):35-7.
9. Rantz MJ, Flesner MK, Zwygart-Stauffacher M. Improving care in nursing homes using quality measures/indicators and complexity science. Journal of Nursing Care Quality. 2010;25(1):5-12.
10. Shoffner J, Wilson KM. MDS quality indicators: looking beneath the numbers. Journal of the American Medical Directors Association. 2002;3(4):H40-1.
11. Stefanacci RG, Riddle A. Delivering on quality measures: Six new CMS SNF quality measures. Geriatric Nursing. 2016;37(5):398-400.
12. Steventon A. Making the best use of administrative data. BMJ (Online). 2013;346(7897):f1284.
13. Stryer D, Clancy C. Boosting performance measure for measure. British Medical Journal. 2003;326(7402):1278.
