# Supplementary material for: Evidence for publicly reported quality indicators in residential long-term care: a systematic review
Source: BMC Health Serv Res. 2022 Nov 24;22:1408. doi: 10.1186/s12913-022-08804-7 (PMC9686098; doi:10.1186/s12913-022-08804-7)
Supplement: Supplementary file 5 — Additional file 5. Reported measurement properties on validity and reliability per QI. [file 12913_2022_8804_MOESM5_ESM.docx]

**Additional file 5: Reported measurement properties on validity and reliability per quality indicator.**

| **Quality indicator (QI)** | **Reporting countries** | **Assessment by stakeholders**^a^ | **Interrater reliability**  **(Kappa**^b^**)** | **Validity**  **(currently-measured QI)** | **Other evidence**^d^ |
| --- | --- | --- | --- | --- | --- |
| **Validity and reliability available for the QI** (sorted by a) validity and b) interrater reliability values) | | | | | |
| Pressure ulcer prevalence | U.S.*,  New Zealand,  Australia,  Belgium, Netherlands,  Sweden | 3 (U.S.)  2 (New Zealand)  4 (Australia)  3 (Belgium)  2 (Netherlands)  -- (Sweden) | .74 (1) (U.S.)  .97 (2) (Netherlands) | High (1)  (U.S.^c^) | Prevalence QI (stage 1-4) showed differences in resident outcomes between NHs (3)  No difference in care processes between NHs with high and low prevalence (4)  High sensitivity for nursing practice according to experts (5)  High validity (1) |
| Antipsychotics prevalence | U.S.,  Canada*,  Sweden | 2 (U.S.)  4 (Canada)  2 (Sweden) | .89 (1) | Moderate (6) (Canada) | High sensitivity for the practice of physicians in NHs according to experts (5) |
| Bladder incontinence worsening | Canada*,  New Zealand | -- (Canada)  2 (New Zealand) | .87 (1) | Moderate (6) (Canada) | High validity (1) |
| Pain worsening | Canada*,  New Zealand | -- (Canada)  2 (New Zealand) | .73 (1) | Moderate (6) (Canada) | High sensitivity for nursing practice according to experts (5)  Moderate validity (1) |
| Fall prevalence | Canada*,  New Zealand,  Belgium,  Australia,  Sweden | 4 (Canada)  2 (New Zealand)  3 (Belgium)  4 (Australia)  -- (Sweden) | .52 (1) | Moderate (6) (Canada) | Showed differences in resident outcomes between NHs (3)  34%/53% sensitivity of QI falls in last 30/180 days and 97%/97% specificity. The resident documentation showed significantly more falls than the MDS (7)  Moderate validity (1) |
| Pressure ulcer worsening | Canada*,  New Zealand | -- (Canada)  2 (New Zealand) | .74 (1) | Not valid (6)  (Canada) | High sensitivity for nursing practice according to experts (5)  Not valid (1) |
| Behavioral symptoms worsening | Canada*,  New Zealand | -- (Canada)  2 (New Zealand) | .72 (1) | Not valid (6) (Canada) | Not valid (1) |
| Depression signs worsening | Canada*,  New Zealand | 4 (Canada)  2 (New Zealand) | .60 (1) | Not valid (6)  (Canada) | Moderate validity (1) |
| **Only validity available for the QI** (sorted by validity) | | | | | |
| Mid-loss ADL worsening | Canada*,  New Zealand | 4 (Canada)  2 (New Zealand) | -- | Moderate (6) (Canada) | -- |
| Mid-loss ADL improvement | Canada*,  New Zealand | 4 (Canada)  2 (New Zealand) | -- | Moderate (6) (Canada) | -- |
| Behavioral symptoms improvement | Canada*,  New Zealand | 4 (Canada)  2 (New Zealand) | -- | Moderate (6) (Canada) | -- |
| New pressure ulcer development | Canada*,  New Zealand | -- (Canada)  2 (New Zealand) | -- | Not valid (6)  (Canada) | High sensitivity for nursing practice according to experts (5) |
| **Only reliability available for the QI** (sorted by interrater reliability) | | | | | |
| Bowel incontinence worsening | New Zealand | 2 | .88 (1) |  | Moderate validity (1) |
| Late-loss ADL worsening | U.S.  New Zealand | 2 (U.S.)  2 (New Zealand) | .84 (1) |  | Showed differences in resident outcomes between NHs (3)  High validity (1) |
| Communication worsening | New Zealand | 2 | .83 (1) | -- | Moderate validity (1) |
| ADL-long form  worsening | New Zealand | 2 | .83 (1) | -- | High validity (1)  Nursing homes have little influence on ADL changes (8) |
| Locomotion worsening | U.S.*  New Zealand | -- (U.S.)  2 (New Zealand) | .82 (1) | -- | High validity (1) |
| Feeding tube | New Zealand | 2 | .80 (1) |  | Moderate validity (1) |
| Cognitive ability worsening | New Zealand | 2 | .76 (1) | -- | Moderate validity (1) |
| Bladder catheter prevalence | U.S.*  New Zealand | 3 (U.S.)  2 (New Zealand) | .71 (1) | -- | High sensitivity for the practice of physicians in Nhs according to experts (5)  High validity (1) |
| Urinary tract infection prevalence | U.S.  New Zealand | 3 (U.S.)  2 (New Zealand) | .53 (1) | -- | Showed differences in resident outcomes between NHs (3)  Sensitivity 57.9%, specificity 86.5%, only 13.9% of residents with urinary tract infections reported in the MDS actually had it (9)  High validity (1) |
| **Neither reliability nor validity available for the QI** (sorted by a) available evidence, b) number of countries using the QI, c) stakeholder involvement) | | | | | |
| Falls with major injury | U.S. | 3 | -- | -- | 40% sensitivity and 93% specificity compared with chart review (10)  62.9% of cases identified in claims data were reported in MDS data for long-stay residents (11) |
| Emergency department visits | U.S.*,  Canada | -- (U.S.)  4 (Canada) | -- | -- | -- |
| Polypharmacy | Australia,  Sweden | 4 (Australia)  -- (Sweden) | -- | -- | -- |
| Advance care planning | Belgium, Netherlands | 3 (Belgium)  2 (Netherlands) | -- | -- | -- |
| Medication review | Netherlands, Norway | 2 (Netherlands)  1 (Norway) | -- | -- | -- |
| Fall-related fractures | Australia | 4 | -- | -- | -- |
| Pressure ulcers new or worsened | Canada* | 4 | -- | -- | -- |
| Medication errors | Belgium | 3 | -- | -- | -- |
| Death in NH | Belgium | 3 | -- | -- | -- |
| Influenza vaccination | U.S. | 3 | -- | -- | -- |
| Pneumococcal vaccination | U.S. | 2 | -- | -- | -- |
| Communication improvement | New Zealand | 2 | -- | -- | -- |
| Cognitive ability improvement | New Zealand | 2 | -- | -- | -- |
| Ability to locomote improvement (from ADL) | New Zealand | 2 | -- | -- | -- |
| Early-loss ADL worsening | New Zealand | 2 | -- | -- | -- |
| Early-loss ADL improvement | New Zealand | 2 | -- | -- | -- |
| Late-loss ADL  improvement | New Zealand | 2 | -- | -- |  |
| Bladder continence improvement | New Zealand | 2 | -- | -- | -- |
| Bowel continence improvement | New Zealand | 2 | -- | -- | -- |
| Toileting plan | Netherlands | 2 | -- | -- | -- |
| Eating preferences | Netherlands | 2 | -- | -- | -- |
| Antianxiety or hypnotic drug use | U.S. | 2 | -- | -- | -- |
| Inappropriate medication | Sweden | 2 | -- | -- | -- |
| Psychotropic drugs | Sweden | 2 | -- | -- | -- |
| Nosocomial infections | Norway | 1 | -- | -- | -- |
| Nutritional assessment | Norway | 1 | -- | -- | -- |
| Receiving antibiotics | Norway | 1 | -- | -- | -- |
| Medical examination in the last 12 months | Norway | 1 | -- | -- | -- |
| Dental examination in the last 12 months | Norway | 1 | -- | -- | -- |
| Hours of medical treatment per week | Norway | 1 | -- | -- | -- |
| Hospitalizations | U.S.* | -- | -- | -- | -- |
| Oral health problems | Sweden | -- | -- | -- | -- |
| Malnutrition | Sweden | -- | -- | -- | -- |
| Risk prevention measures  for pressure ulcers, malnutrition, falls, oral health problems and bladder disfunction | Sweden | -- | -- | -- | -- |
| Use of antibiotics | Sweden | -- | -- | -- | -- |
| Healthcare-associated infections | Sweden | -- | -- | -- | -- |
| Use of pressure-relieving surfaces | Sweden | -- | -- | -- | -- |

Abbreviations: ADL: Activities of Daily Living, QI: Quality indicator, MDS: Minimum Data Set, NH: Nursing home, U.S.: United States

Legend: * The country uses risk-adjustment for the QI; -- No information available.

^a^The involvement of stakeholders was categorized as follows: 1= It is known from the available information that experts were consulted for the development of the QI, or that the QI was assessed by experts, 2 = QI was assessed by experts and the experts/groups of experts are named, e.g., representatives of the authorities, nursing professionals, information technology specialists, 3 = QI was assessed by experts, the experts/expert groups are named and the assessment criteria are clearly defined, e.g., comprehensibility of definition, relevance, feasibility of measurement 4 = QI was assessed by experts, the experts/expert groups are named, the assessment criteria are clearly defined and the results of the assessment is clearly described.

^b^Kappa ≥ .60 = good to very good reliability; .60 > Kappa ≥ .40 = moderate reliability; Kappa < .40 = lacking or weak reliability; For the MDS-based QI, average weighted kappa values are reported, i.e., the kappa value was calculated over all MDS items used to calculate the QI.

^c^In the U.S., pressure ulcers are reported for residents at high risk of pressure ulcers. It is not clear from the available information whether the high-risk group in the study and the QIs currently measured are defined in the same way.

^d^In this column we refer to studies on MDS-based QIs but it is not clear from the available information whether the measurements used in these studies are the same as for the QIs currently reported, e.g., we could not retrieve the appendix with the description of the measurement of the QIs from the validation report by Morris et al., 2003 (1)

**References:**

1. Morris J.N, Moore T JR, Mor V, Angelelli J, Berg K, Hale C, et al. Validation of long-term and post-acute care quality indicators. Cambridge, Massachusetts: Abt Associates Inc, Brown University; 2003.

2. Bours GJ, Halfens RJ, Lubbers M, Haalboom JR. The development of a national registration form to measure the prevalence of pressure ulcers in The Netherlands. Ostomy Wound Manage. 1999;45(11):28-33, 6-8, 40.

3. Rantz MJ, Hicks L, Petroski GF, Madsen RW, Mehr DR, Conn V, et al. Stability and Sensitivity of Nursing Home Quality Indicators. Journals of Gerontology Series A: Biological Sciences & Medical Sciences. 2004;59(1):79-82.

4. Bates-Jensen BM, Cadogan M, Osterweil D, Levy-Storms L, Jorge J, Al-Samarrai N, et al. The Minimum Data Set Pressure Ulcer indicator: does it reflect differences in care processes related to pressure ulcer prevention and treatment in nursing homes? Journal of the American Geriatrics Society. 2003;51(9):1203-12.

5. Estabrooks CA, Knopp-Sihota JA, Norton PG. Practice sensitive quality indicators in RAI-MDS 2.0 nursing home data. BMC research notes. 2013;6:460.

6. Jones RN, Hirdes JP, Poss JW, Kelly M, Berg K, Fries BE, et al. Adjustment of nursing home quality indicators. BMC health services research. 2010;10:96.

7. Hill-Westmoreland EE, Gruber-Baldini AL. Falls Documentation in Nursing Homes: Agreement Between the Minimum Data Set and Chart Abstractions of Medical and Nursing Documentation. Journal of the American Geriatrics Society. 2005;53(2):268-73.

8. Phillips CD, Shen R, Chen M, Sherman M. Evaluating Nursing Home Performance Indicators: An Illustration Exploring the Impact of Facilities on ADL Change. Gerontologist. 2007;47(5):683-9.

9. Stevenson KB, Moore JW, Sleeper B. Validity of the Minimum Data Set in identifying urinary tract infections in residents of long-term care facilities. Journal of the American Geriatrics Society. 2004;52(5):707-11.

10. Mintz J, Lee A, Gold M, Hecker EJ, Colón‐Emeric C, Berry SD. Validation of the Minimum Data Set Items on Falls and Injury in Two Long‐Stay Facilities. Journal of the American Geriatrics Society. 2021;69(4):1099-100.

11. Sanghavi P, Pan S, Caudry D. Assessment of nursing home reporting of major injury falls for quality measurement on nursing home compare. Health Serv Res. 2020;55(2):201-10.
